# Genome-wide identification and expression analysis of the BURP domain-containing genes in *Gossypium hirsutum*

**DOI:** 10.1186/s12864-019-5948-y

**Published:** 2019-07-08

**Authors:** Huiru Sun, Hengling Wei, Hantao Wang, Pengbo Hao, Lijiao Gu, Guoyuan Liu, Liang Ma, Zhengzheng Su, Shuxun Yu

**Affiliations:** 1 State Key Laboratory of Cotton Biology, Institute of Cotton Research of CAAS, Anyang, 455000 China; 2 College of Agronomy, Northwest A&F University, Yangling, 712100 China

**Keywords:** *Gossypium* spp., BURP domain, Promoter, Expression patterns, Abiotic stress

## Abstract

**Background:**

Many BURP domain-containing proteins, which are unique to plants, have been identified. They performed diverse functions in plant development and the stress response. To date, only a few BURP domain-containing genes have been studied, and no comprehensive analysis of the gene family in cotton has been reported.

**Results:**

In this study, 18, 17 and 30 putative BURP genes were identified in *G. raimondii* (D_5_), *G. arboreum* (A_2_) and *G. hirsutum* (AD_1_), respectively. These BURP genes were phylogenetically classified into eight subfamilies, which were confirmed by analyses of gene structures, motifs and protein domains. The uneven distribution of BURPs in chromosomes and gene duplication analysis indicated that segmental duplication might be the main driving force of the *GhBURP* family expansion. Promoter regions of all *GhBURPs* contained at least one putative stress-related cis-elements. Analysis of transcriptomic data and qRT-PCR showed that *GhBURPs* showed different expression patterns in different organs, and all of them, especially the members of the RD22-like subfamily, could be induced by different stresses, such as abscisic acid (ABA) and salicylic acid (SA), which indicated that the *GhBURPs* may performed important functions in cotton’s responses to various abiotic stresses.

**Conclusions:**

Our study comprehensively analyzed *BURP* genes in *G. hirsutum*, providing insight into the functions of *GhBURPs* in cotton development and adaptation to stresses.

**Electronic supplementary material:**

The online version of this article (10.1186/s12864-019-5948-y) contains supplementary material, which is available to authorized users.

## Background

Upland cotton, the most widely cultivated cotton in the world, is an important worldwide commercial crop for its natural fiber and edible oil. However, the growth of upland cotton was frequently challenged by diverse environmental stresses (such as drought, salinity and heat) during the whole developmental process which ultimately compromised economic yield [1]. BURP domain-containing proteins are known to play important roles in plant development and stress responses [2–4]. The BURP domain-containing proteins were characterized by the conserved C-terminal domain, which was named based on four typical members: BNM2 (a microspore protein in *Brassica napus*) [5], USP (an unknown seed protein in *Vicia faba*) [6], RD22 (a dehydration-responsive protein in *Arabidopsis thaliana*) [2] and PG1β (a non-catalytic β-subunit of the polygalacturonase isozyme 1 in *Lycopersicon esculentum*) [7]. To date, BURP domain-containing proteins have been found only in plants, indicating that these proteins may have specific functions in plant development.

Generally, BURP domain-containing proteins contained several conserved modules: a putative signal peptide at the N-terminal hydrophobic domain; the BURP domain at the C-terminus, which included several conserved amino acid sites and four repeat cysteine-histidine (CH) motifs, namely, CHX_10_CHX_23-37_CHX_23–26_CHX_8_W (X = any amino acid residue); the variable region in the middle, unique to each BURP protein, which included a short conserved segment or other short segments; and an optional segment consisting of repeated units [8, 9]. According to sequence alignment and phylogenetic analysis of the BURP domain-containing proteins identified in many species, such as soybean [10], poplar [11], alfalfa [12], rice [9], maize and sorghum [13], the members of the BURP family could be divided into BNM2-like, USP-like, RD22-like, PG1β-like and other subfamilies. The main difference among members from different BURP subfamilies was the variable region between the N-terminal hydrophobic and the C-terminal BURP domains. The variable region of the BNM2-like proteins contained only a short conserved segment. The USP-like and RD22-like proteins contained a sequence segment (~ 30 amino acids), while the RD22-like proteins also contained repeat units (~ 20 amino acids) [14]. The PG1β-like proteins contained a sequence segment (14 amino acids) that was distinguished from members of other subfamilies [7].

Many BURP proteins have been identified and classified based on sequence characteristics. However, the members from different subfamilies showed various expression patterns and functions. According to previous studies, BURP genes potentially perform two main functions: maintaining normal plant development and responding to stresses.

In plant development and metabolism, many BURP genes have been isolated and played significant roles in the development of reproductive organs, such as anther, microspore and seed, and in the metabolism of pectin in the cell wall. For instance, the expression of *BNM2* indicated its functions in the development of microspores [5, 15, 16]. VfUSP, a BURP protein found in field bean, might be involved in regulating early development of zygotic embryogenesis [6, 17]. *ASG1*, a BURP gene found in apomictic *Panicum*, probably regulated the formation of aposporous initial cells [18]. OsRAFTIN1, an anther-specific BURP protein in rice, was crucial for pollen development [19]. The expression pattern of *RA8*, another anther-specific BURP gene in rice indicated its importance for dehiscence of anther [3]. SCB1, a seed coat-specific BURP protein isolated from soybean, might regulate the differentiation of parenchyma cells in seed coat [20]. AtUSP1, a BURP protein in *Arabidopsis*, was synthesized in cellular compartments, indicating a significant function in seed development [21]. PG1β, a non-catalytic β-subunit of the PG1 complex in tomato, might be essential for pectin metabolism by regulating pectin solubilization and degradation [7, 22]. These reports showed the clues of BURPs’ functions in various organs development in plants.

In response to stress treatments, some BURP proteins mainly from the RD22-like and BNM2-like subfamilies have been reported to be stress-induced. *RD22*, a reference gene for drought stress in *Arabidopsis*, was also induced by salt and abscisic acid (ABA) [2, 23]. *AtUSP1* was also demonstrated as a suppressor of the ABA-mediated moisture stress response. *BgBDC1*, *2*, *3* and *4*, four BURP genes homologous to *AtRD22* in the mangrove, responded to biotic stresses and abiotic stresses in an ABA-mediated manner [24]. *BnBDC1*, a BURP gene with sequence similarity to *AtRD22* in oilseed rape, was induced by various stresses, including salt, osmotic and UV irradiation, and plant hormones, such as ABA and SA [25]. *SBIP-355*, a BURP gene homologous to *AtRD22* in tobacco, might be involved in plant defense through the SA pathway [26]. *Sali5-4a* and *Sali3–2*, two BURP genes found in soybean, were induced by aluminum and *Sali3–2* was involved in the salt tolerance [27–29]. Moreover, comprehensive analyses of *BURP* family genes in many plants showed that most of them were responsive to stress treatments [9, 10, 12, 13]. These results implied that *BURP* genes performed crucial functions not only in plant development but also in response to abiotic stresses and might be involved in phytohormone signal pathways, such as ABA or SA.

At present, only one *BURP* gene in cotton (*GhRDL1*) has been cloned and could interact with *GhEXPA1*, and their co-expression could strikingly increase bolls and fruit production [30]. Meanwhile, *GhRDL1* and *GhEXPA1*, as target genes of two HD-ZIP IV transcription factors, *GhHOX3* and *GhHD1*, could also influence fiber length finally [31]. These findings indicated that BURP genes might perform important functions in cotton. However, most of the BURP genes in cotton are unknown, and no available genome-wide analyses of the cotton BURP gene family have been reported. So, it is considerably interesting to identify and comprehensively analyze BURP family in upland cotton. In this study, putative *BURP* genes were identified by screening the genome sequence of *Gossypium raimondii*, *Gossypium arboreum* and allotetraploid cultivated cotton (*Gossypium hirsutum*) [32–36]. The comprehensive analyses of *BURP* genes in three cotton species, including gene structure, phylogenetic tree and expression characteristics in different tissues and under various stress treatments will provide a foundation for further studies on the potential functions of BURPs in cotton growth and stress response.

## Results

### Identification of BURP family members in *G. raimondii*, *G. arboreum* and *G. hirsutum*

To identify BURP family genes, the BURP domain (PF03181) was served as a query to search against the protein databases of *G. raimondii*, *G. arboreum and G. hirsutum*. A total of 18, 17 and 30 putative BURP proteins were identified in *G. raimondii, G. arboreum* and *G. hirsutum*, respectively, which were confirmed to contain the conserved BURP domain by Pfam and SMART databases. Sixty-four putative BURP proteins were named according to their previously reported homologous groups in other species. The length of putative GrBURP protein sequences ranged from 212 amino acids (aa) (GrBURP2) to 649 aa (GrPG1); GaBURPs ranged from 220 aa (GaBURP3 and GaBURP4) to 649 aa (GaPG1), and GhBURPs ranged from 166 aa (GhBURP5) to 733 aa (GhBNM4). The predicted molecular weight (Mw), isoelectric point (pI), grand average (GRAVY) of hydropathicity and subcellular localization of these proteins were shown in Table 1.

**Table 1:** The *BURP* genes in three cotton species

ID	Name	Length of amino acids	Molecular weight/kDa	Theoretical pI	Grand average cof hydropathicity	Predicted subcellular localization	Chromosome number	Location
Ga03G2725	GaBNM1	281	31.42	7.6	0.046	Plasma Membrane	Chr03	135165245–135166171(+)
Ga04G0288	GaBNM2	298	33.34	5.94	−0.189	Extracellular	Chr04	3515535–3516509(+)
Ga08G0770	GaBNM3	472	53.09	5.66	−0.45	Nuclear	Chr08	12701857–12704664(+)
Ga03G2726	GaBNM4	285	31.47	8.66	0.233	Plasma Membrane	Chr03	135167409–135168579(+)
Ga07G1165	GaBNM5	599	67.33	5.99	−0.651	Cytoplasmic(1.355) /Nuclear(1.514)	Chr07	18163166–18165657(+)
Ga10G0842	GaBNM6	397	44.68	5.97	−0.338	Cytoplasmic	Chr10	17979735–17981015(−)
Ga13G0509	GaBNM7	277	31.26	7.64	0.144	Plasma Membrane	Chr13	6572148–6573084(−)
Ga05G0755	GaBURP1	305	34.26	6.83	−0.085	Extracellular	Chr05	6608432–6613461(−)
Ga05G0756	GaBURP2	186	20.32	6.05	0.131	Extracellular(1.412) /Plasma Membrane(1.195) /Cytoplasmic(1.317)	Chr05	6615692–6631332(−)
Ga08G0163	GaBURP3	220	24.89	9.28	−0.148	Plasma Membrane(1.255)/ Mitochondrial(1.673)	Chr08	1266719–1267381(+)
Ga08G0165	GaBURP4	220	24.79	9.39	−0.13	Plasma Membrane(1.274)/ Mitochondrial(1.775)	Chr08	1275432–1276094(+)
Ga08G0166	GaBURP5	482	52.61	7.6	−0.426	Extracellular(1.491) /Nuclear(1.126)	Chr08	1284145–1287039(+)
Ga13G2660	GaBURP6	243	26.95	9.05	−0.315	Cytoplasmic(1.095) /Mitochondrial(1.053) /Nuclear(1.020)	Chr13	122516969–122517700(+)
Ga03G1868	GaPG2	609	66.63	6.23	−0.52	Extracellular	Chr03	120728911–120730947(+)
Ga01G1594	GaPG3	604	66.39	6.94	−0.52	Extracellular	Chr01	65377300–65380623(+)
Ga03G1340	GaPG1	649	71.18	9.01	−0.437	Extracellular(1.520) /Vacuole(1.353)	Chr03	80058785–80060850(+)
Ga05G0527	GaRD1	335	36.56	6.64	−0.268	Extracellular(1.555) /Cytoplasmic(1.749)	Chr05	4680805–4689016(+)
Gorai.005G261700.1	GrBNM1	281	31.47526	8.08	−0.097	Cytoplasmic	Chr05	63641010–63642551(+)
Gorai.012G152100.1	GrBNM2	298	33.30303	5.99	−0.201	Periplasmic(1.851) /Cytoplasmic(1.347)	Chr12	32624207–32625595(−)
Gorai.004G078900.1	GrBNM3	472	52.84154	5.62	−0.494	OuterMembrane	Chr04	9346335–9348788(+)
Gorai.005G261800.1	GrBNM4	238	26.04937	8.91	0.017	Periplasmic	Chr05	63644230–63645258(+)
Gorai.001G119700.1	GrBNM5	515	57.90174	5.83	−0.514	Extracellular(1.226) /Periplasmic(1.798)	Chr01	14537465–14539428(+)
Gorai.011G215100.1	GrBNM6	397	44.7685	6.03	−0.327	Periplasmic	Chr11	51614479–51617258(+)
Gorai.013G053000.1	GrBNM7	277	31.26245	8.1	0.114	InnerMembrane(1.706) /Cytoplasmic(2.014)	Chr13	4975454–4976764(−)
Gorai.009G074500.1	GrBURP1	313	35.32591	8.58	−0.13	Periplasmic(1.816) /Cytoplasmic(2.181)	Chr09	5341346–5342757(−)
Gorai.009G074600.1	GrBURP2	212	23.94261	5.69	−0.075	Cytoplasmic	Chr09	5343679–5344657(−)
Gorai.009G074700.1	GrBURP3	256	28.12751	7.61	−0.118	Periplasmic	Chr09	5350696–5351800(−)
Gorai.004G015400.1	GrBURP4	220	24.78878	9.18	−0.15	Cytoplasmic	Chr04	1118786–1119620(+)
Gorai.004G015500.1	GrBURP5	276	30.39973	8.95	−0.274	Extracellular(1.393) /OuterMembrane(1.389) /Periplasmic(1.366)	Chr04	1126045–1127011(+)
Gorai.013G252700.1	GrBURP6	243	26.91194	8.85	−0.314	OuterMembrane(1.167) /Periplasmic(1.647) /Cytoplasmic(1.550)	Chr13	57021536–57022390(+)
Gorai.005G136300.1	GrPG1	649	71.54197	9.01	−0.428	OuterMembrane	Chr05	35061399–35063863(+)
Gorai.005G182600.1	GrPG2	628	68.66988	7.22	−0.514	OuterMembrane	Chr05	53449903–53452254(+)
Gorai.002G161400.1	GrPG3	616	67.72389	7.96	−0.511	OuterMembrane	Chr02	36980855–36983098(+)
Gorai.009G052100.1	GrRD1	396	42.13522	9.19	−0.326	Periplasmic	Chr09	3755513–3757859(+)
Gorai.009G052200.1	GrRD2	367	40.09897	6.36	−0.258	Periplasmic	Chr09	3760180–3772019(+)
Gh_A03G1881	GhBNM1	281	31.34713	8.37	−0.063	Cytoplasmic	A03	99771377–99772299(+)
Gh_D02G2319	GhBNM2	281	31.50228	8.08	−0.107	Cytoplasmic	D02	66842483–66843403(+)
Gh_A04G1039	GhBNM3	298	33.32507	5.94	−0.187	Periplasmic(1.909) /Cytoplasmic(1.301)	A04	60430883–60431857(−)
Gh_A08G0606	GhBNM4	733	81.84821	5.74	−0.661	OuterMembrane	A08	11071560–11073866(+)
Gh_A07G0976	GhBNM5	547	61.31128	5.79	−0.607	Extracellular(1.490)/Extracellular(1.357) /Cytoplasmic(1.067)	A07	18492640–18494375(+)
Gh_D07G1054	GhBNM6	567	63.70206	5.9	−0.591	Extracellular(1.496) /Periplasmic(1.625)	D07	14895486–14899357(+)
Gh_A10G1660	GhBNM7	252	28.01502	6.36	−0.094	Periplasmic(2.091) /Cytoplasmic(1.920)	A10	88329967–88330725(+)
Gh_D10G1918	GhBNM8	252	28.07105	6.48	−0.113	Periplasmic(2.080) /Cytoplasmic(1.909)	D10	53448375–53449133(+)
Gh_D13G0464	GhBNM9	277	31.26245	8.1	0.114	InnerMembrane(1.706) /Cytoplasmic(2.014)	D13	5415661–5416581(−)
Gh_A05G0583	GhBUPR1	313	35.26984	8.57	−0.136	Periplasmic(1.899) /Cytoplasmic(2.139)	A05	6266697–6267846(−)
Gh_D05G0713	GhBUPR2	313	35.29288	8.57	−0.127	Periplasmic(2.076) /Cytoplasmic(1.899)	D05	5850057–5851213(−)
Gh_A05G0584	GhBUPR3	212	23.86255	5.69	−0.074	Cytoplasmic	A05	6268879–6269517(−)
Gh_D05G0714	GhBUPR4	212	23.91258	5.88	−0.092	Cytoplasmic	D05	5852228–5852866(−)
Gh_A05G0585	GhBUPR5	166	18.02802	6.57	0.224	Periplasmic	A05	6274388–6275502(−)
Gh_A08G2518	GhBUPR6	220	24.83185	9.28	−0.145	Periplasmic(1.731) /Cytoplasmic(2.249)	scaffold2270_A08	37419–38081(+)
Gh_D08G2754	GhBUPR7	220	24.67866	9.04	−0.106	Periplasmic(1.664) /Cytoplasmic(2.128)	scaffold4315_D08	128279–128941(+)
Gh_A08G2519	GhBUPR8	482	52.57868	6.56	−0.414	OuterMembrane	scaffold2270_A08	42942–45824(+)
Gh_D08G2755	GhBUPR9	276	30.33856	8.16	−0.261	Extracellular(1.170) /OuterMembrane(1.349) /Periplasmic(1.450)	scaffold4315_D08	133781–135310(+)
Gh_A13G1887	GhBUPR10	243	26.92202	9.04	−0.312	Periplasmic	A13	78553086–78553817(+)
Gh_D13G2274	GhBUPR11	243	26.86994	9.05	−0.313	Periplasmic(1.924) /Cytoplasmic(1.410)	D13	58789956–58790687(+)
Gh_A03G0838	GhPG1	649	71.20247	9.06	−0.447	OuterMembrane	A03	45353566–45355631(+)
Gh_D02G1172	GhPG2	649	71.38377	9.07	−0.431	OuterMembrane	D02	35492312–35494383(−)
Gh_A03G1222	GhPG3	628	68.85208	6.74	−0.509	OuterMembrane	A03	86599810–86601849(+)
Gh_D02G1656	GhPG4	628	68.76397	6.94	−0.515	OuterMembrane	D02	57114338–57116390(+)
Gh_D01G2373	GhPG5	616	67.78298	7.96	−0.508	OuterMembrane	scaffold3762_D01	162083–164005(−)
Gh_Sca004926G01	GhPG6	616	67.97921	6.47	−0.507	OuterMembrane	scaffold4926	17075–18997(+)
Gh_A05G0390	GhRD1	376	40.25604	8.85	−0.299	Periplasmic	A05	4358119–4359765(+)
Gh_D05G0507	GhRD2	376	40.22106	9.01	−0.304	Periplasmic	D05	4071766–4073401(+)
Gh_A05G0391	GhRD3	335	36.60908	6.89	−0.267	Periplasmic	A05	4369019–4370547(+)
Gh_D05G0508	GhRD4	335	36.49686	6.89	−0.283	Periplasmic	D05	4091376–4092898(+)

*AA* amino acid, Molecular weight, *pI* Isoelectric point, *GRAVY* Grand average of hydropathicity

### Phylogenetic analysis and classification of the BURP gene family

**Fig. 1:**
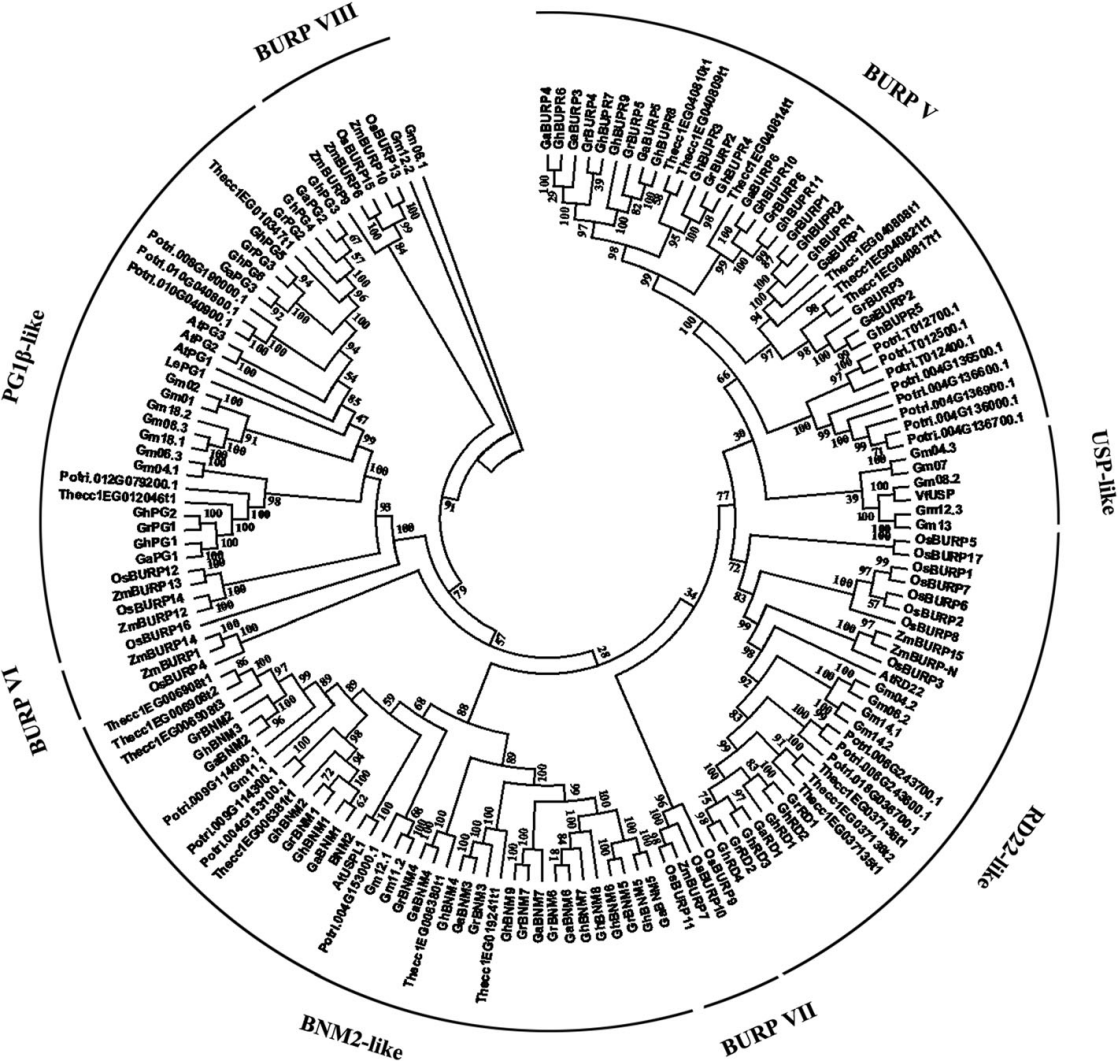
Phylogenetic tree of BURP proteins. The predicted crotein sequences from *Oryza sativa* (17), *Zea mays* (10), *Arabidopsis thaliana* (4), *Glycine max* (21), *Populus trichocarpa* (19), *Theobroma cacao* (17), *G. raimondii* (18), *G. arboreum* (17), *G. hirsutum* (30) and 4 host BURP proteins (AtRD22, VfUSPs, BNM2 and Le-PG1β) were aligned with ClustalX 2.0, and the phylogenetic tree was constructed by MEGA 6.0 with the neighbor-joining (NJ) method

To investigate the evolutionary relationships of BURP proteins, phylogenetic analysis of 157 predicted BURP proteins from *Oryza sativa* (17), *Zea mays L* (10), *Arabidopsis thaliana* (4 and the remaining one, AtRD22, included in the 4 host BURP proteins), *Glycine max* (21), *Populus trichocarpa* (19), *Theobroma cacao* (17), *G. raimondii* (18), *G. arboreum* (17), *G. hirsutum* (30) and 4 host BURP proteins (AtRD22, VfUSPs, BNM2 and Le-PG1β) were used to construct a phylogenetic tree. The different number of BURP members in *Zea mays* (15 to 10), *Populus trichocarpa* (18 to 19) and *Glycine max* (23 to 21) between our study and previous reports might result from choosing only one transcript of the gene and choosing different genomic versions. These BURP proteins were clustered into eight subfamilies (BNM2-like, USP-like, RD22-like, PG1β-like, BURP V, BURP VI, BURP VII and BURP VIII) (Fig. 1 and Additional file 1: Table S1). The BURP VI and BURP VII subfamilies contained only the BURP members from two monocots, *Oryza sativa* and *Zea mays*. The BNM2-like, BURP V and USP-like subfamilies contained only the BURP members from the investigated dicots. The results indicated that BURP proteins of these subfamilies might evolve in different directions and show diverse functions in different plants. Notably, BURP proteins from *Populus trichocarpa*, *Theobroma cacao*, *G. raimondii*, *G. arboreum* and *G. hirsutum* showed similar distributions in four subfamilies (BNM2-like, PG1β-like, RD22-like and BURP V).

### Chromosomal location, gene duplication event and syntenic analysis of *BURP* genes

**Fig. 2:**
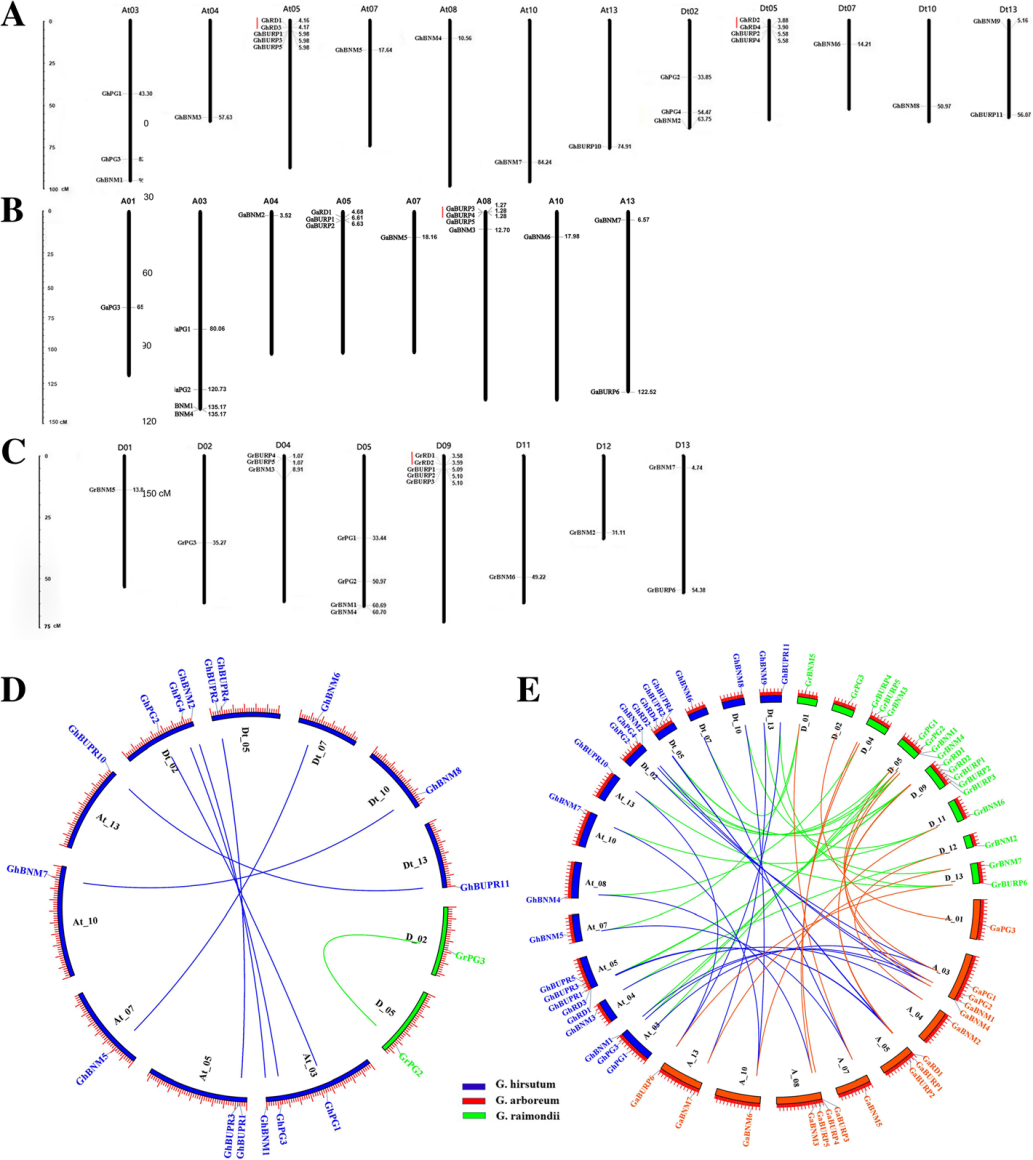
Chromosomal location, gene duplication events and syntenic anaylsis of *BURPs* in three cotton species. The putative *BURPs* are shown on the different chromosomes. **a**, **b** and **c** represent the physical locations of *BURPs* in *G. hirsutum* (**a**), *G. arboreum* (**b**) and *Gossypium raimondii* (**c**), respectively. The scale bar represents megabases (Mb). The chromosome numbers are indicated on the top of each bar. Red lines represent the gene pairs of tandem duplication. **d**-**e** Gene pairs of segment duplication and syntenic relationships among *GrBURPs*, *GaBURPs* and *GrBURPs*. The Circos tool was used to draw the genome visualization. Green, red and blue are filled in chromosomes of *Gossypium raimondii*, *G. arboreum* and *G. hirsutum*, respectively. The corresponding color lines represent gene pairs of segment duplication in three cotton species

According to the genomic location of 65 *BURP* genes, the chromosomal distributions of *GrBURPs*, *GaBURPs* and *GhBURPs* were shown in Table 1 and Fig. 2. In *G. raimondii*, 18 *GrBURPs* were unevenly anchored on eight chromosomes. D05 and D09 contained four and five *GrBURPs*, respectively. The other six chromosomes, D01, D02, D04, D11, D12 and D13, contained one to three *GrBURPs* (Fig. 2c). In *G. arboreum*, 17 *GaBURPs* were located on eight chromosomes. A03 and A08 contained the most *GaBURPs* (4), while the other six chromosomes contained only one to three *GaBURPs* (Fig. 2b). In *G. hirsutum*, a total of 24 *GhBURPs* were unevenly mapped to twelve chromosomes, while the other six *GhBURPs* were located on unassembled scaffolds. At05 and Dt05 contained five and four *GhBUPRs*, respectively. The other ten chromosomes contained only one to three *GhBURPs* (Fig. 2a).

Gene duplication events, including segment duplication and tandem duplication have been studied for their vital role in genomic evolution and formation of gene family [37]. To explore the relationships between BURP genes and gene duplications in cotton, gene duplication analysis was performed and shown in Fig. 2 and Table 2. In *G. arboreum*, one tandem duplication event (*GaBURP3*/*GaBURP4*) was identified. In *G. raimondii*, two gene duplication events were discovered, including one segment duplication (*GrPG2* / *GrPG3*) and one tandem duplication event (*GrRD1* / *GrRD2*). In *G. hirsutum*, 13 gene duplication events, including 11 segment duplications and 2 tandem duplications (*GhRD1* / *GhRD3*, *GhRD2*/ *GhRD4*), accounted for 86.67% of the *GhBURP* gene family. It is noteworthy that the segmental duplication pairs identified in *G. hirsutum* are all orthologs from A and D subgenomes, indicating that the hybridization of A and D genomes and following tetraploidization led to the duplication of these genes.

**Table 2:** Gene duplication, Ka/Ks and divergence times of gene duplicated *BURP* gene pairs in three cotton species

Duplication gene 1	Duplication gene 2	Identity	Duplicate type	Ks	Ka	Ka/Ks	Purifying selection	Age/MYA
GaBURP3	GaBURP4	94.55	tandem	0.0424	0.0239	0.5764	Yes	8.15
GrPG3	GrPG2	80.84	segment	0.6863	0.1050	0.1530	Yes	263.96
GrRD1	GrRD2	96.12	tandem	0.0498	0.0205	0.4116	Yes	19.15
GhPG2	GhPG1	96.76	segment	0.0615	0.0142	0.2309	Yes	23.65
GhPG4	GhPG3	98.89	segment	0.0325	0.0049	0.1508	Yes	12.50
GhPG5	GhPG6	97.4	segment	0.0260	0.0113	0.4346	Yes	10.00
GhBUPR2	GhBUPR1	97.44	segment	0.0684	0.0111	0.1623	Yes	26.31
GhBUPR11	GhBUPR10	94.65	segment	0.0424	0.0235	0.5542	Yes	16.31
GhBUPR4	GhBUPR3	96.23	segment	0.0353	0.0184	0.5212	Yes	13.58
GhBUPR7	GhBUPR6	95.91	segment	0.0443	0.0188	0.4244	Yes	17.04
GhBNM5	GhBNM6	94.65	segment	0.0683	0.0295	0.4319	Yes	26.27
GhBNM7	GhBNM8	96.03	segment	0.0414	0.0173	0.4179	Yes	15.92
GhBNM2	GhBNM1	97.51	segment	0.0147	0.0126	0.8571	Yes	5.65
GhRD1	GhRD3	92.84	tandem	0.0591	0.0326	0.5516	Yes	22.73
GhRD2	GhRD4	95.82	tandem	0.0542	0.0218	0.4022	Yes	20.85

*Ka* nonsynonymous substitution rate, *Ks* synonymous substitution rate, *MYA* million years ago

The selection pressure of *BURP* gene duplication pairs during their evolution was investigated by analysis of non-synonymous (Ka) substitution / synonymous (Ks) substitution ratio. All of the Ka/Ks ratios were less than 1.0 which implied that these gene pairs have evolved mainly under purifying selection after gene duplication events (Table 2). With restriction of divergence by purifying selection, gene duplication pairs might exert analogous functions. Moreover, the divergence times between the gene duplication pairs were calculated (Table 2). In *G. raimondii* and *G. arboreum*, the divergence times of two tandem duplication BURP pairs were inferred to be 8 and 19 million years ago (MYA), while the divergence time of the segmentally duplicated *BURP* gene pair was inferred to be ~ 264 MYA. In *G. hirsutum*, the divergence times of the duplicated *BURP* pairs were presumed to be ~ 5.65 to 26.30 MYA, with an average of 15.57 MYA.

According to syntenic analysis, 30, 26 and 18 homologous gene pairs were found between *G. hirsutum* and *G. raimondii*, *G. hirsutum* and *G. arboreum*, and *G. raimondii* and *G. arboretum*, respectively (Fig. 2e). Meanwhile, both thirteen *GrBURPs* and *GaBURPs* had orthologous *GhBURPs* (Additional file 2: Table S2), whereas the orthologs of the other five *GrBURPs* and four *GaBURPs* in upland cotton were lost. The results revealed that the *GhBURPs* might experience genic sequence losses during the formation of upland cotton approximately 1–2 MYA [36].

### Sequence analysis of BURP proteins

The signal peptides on the N-terminus and BURP domains on the C-terminus of BURP proteins in three cotton species were searched, and the location information was shown in Additional file 3: Table S3. The results showed that all 65 BURP proteins contained the BURP domain, and 37 BURP proteins contained the signal peptides: 8, 12 and 17 BURPs from *G. arboreum*, *G. raimondii* and *G. hirsutum*, respectively (Additional file 4: Figure S1). The multiple sequence alignment analysis of these BURP proteins displayed several highly conserved amino acid sites and four CH motifs indicating that these sites were important for the basic function of the BURP family (Additional file 5: Figure S2). Interestingly, an aspartic acid (D) was found among all 65 BURP proteins between the last two CH motifs revealing that this site was highly conserved and might be significant for maintaining the basic function of the BURP members in cotton. However, GrBURP3 had low similarity to other proteins due to the lack of the second CH and the variable fourth CH. In general, the C-terminus of BURP proteins in cotton could be summarized as CHX_3_YX_6_CHX_23–28_CHXDX_18-23_CHX_8_W with higher conserved sequences between the last two CH motifs.

### Gene structure and conserved motifs of BURPs in three cotton species

**Fig. 3:**
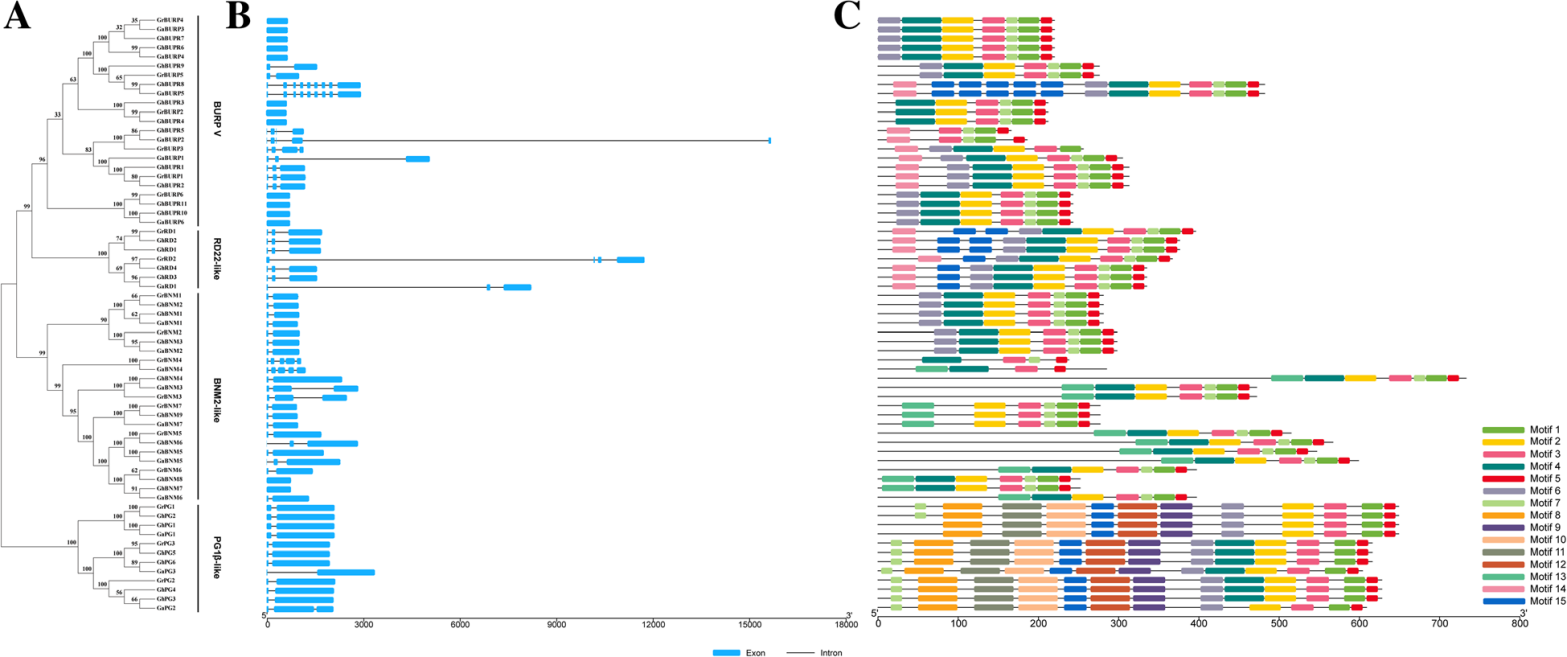
Phylogenetic relationships, exon-intron structure and motif analysis of GrBURPs, GaBURPs and GrBURPs. **a** Phylogenetic analysis of 65 BURP proteins and subfamilies of these proteins using MEGA 6.0 with the neighbor-joining (NJ) method is shown. **b** Exon-intron structure of 65 *BURP* genes. Exons and introns are indicated with green filled boxes and black lines, respectively. **c** The 15 putative motifs of 65 BURP proteins were determined by MEME. Different motifs are represented by different colour boxes

To obtain further insight into the conservation and diversification of the BURPs in three cotton species, analysis of their exon-intron structures and conserved motifs was carried out (Fig. 3 and Additional file 6: Table S4). Most (57/65) of the *BURPs* contained less than 3 exons, except *GrRD2*, *GrBURP3*, *GhBURP5*, *GaBURP2*, *GaBNM4*, *GrBNM4*, *GaBURP5* and *GhBURP8* which contained 4, 5 and 9 exons, respectively. Major members from the PG1β-like and BNM2-like subfamilies contained 2 exons. More than half of the members from the RD22-like and BURP V subfamilies contained 3 and 1 exons, respectively. The results of the gene structure analysis indicated that most of the members from the same subfamilies showed similar gene structures (Fig. 3b).

We further searched 15 conserved motifs using MEME program to obtain more insight into the diversity and similarity of the BURP proteins (Fig. 3c). The BURP domain in the C-terminus contained motif 1, 2, 3, 4, 5, 6 and 7. These seven motifs and their arrangement were similar, especially in the members from the same subfamilies. Closely related BURPs exhibited similar motifs and motif construction. However, motif 13 and motif 8–12 existed only in the BNM2-like and PG1β-like subfamilies, respectively. Motif 14 was present only in the RD22-like and BURP V subfamilies.

### Analysis of cis-elements in putative *GhBURP* promoter regions

**Fig. 4:**
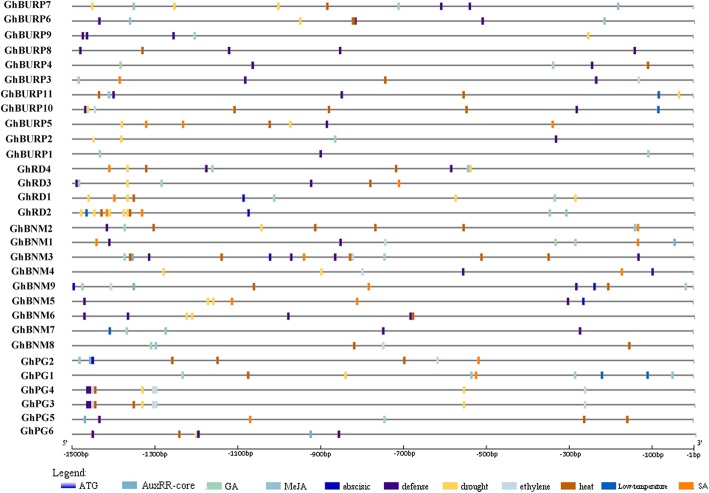
Distribution of major stress-related and plant hormone-related cis-elements in the promoter regions of *GhBURPs*. The location of these cis-elements were confirmed using plantCARE database. Different cis-elements are represented by different colour boxes

Many studies have shown that BURPs participated in various stress responses. To better understand and elucidate the possible regulatory functions of *GhBURPs* under different stresses, we identified putative stress-related and plant hormone-related cis-elements among the 1500 bp promoter regions from the start codons (ATG) of 30 *GhBURPs* (Fig. 4 and Additional file 7: Table S5). Ten kinds of plant hormone-related elements, AuxRR-core (auxin), TGA-element (auxin), P-box (gibberellin), TATC-box (gibberellin), GARE-motif (gibberellin), CGTAC-motif (MeJA), TGACG-motif (MeJA), ERE (ethylene), TCA-element (SA) and ABRE (ABA), and four kinds of stress-responsive regulatory elements, MBS (drought), TC-rich repeats (defense and stress responsiveness), HSE (heat stress) and LTR (cold stress), were identified in the putative promoters of *GhBURPs*. The results revealed that all promoters of 30 *GhBURPs* contained at least one putative stress-responsive elements. The promoters of some *GhBURPs* contained many various stress-responsive elements, such as *GhBNM3* (6 HSEs and 4 TC-rich repeats), *GhRD2* (5 MBSs, 2 HSEs and 1 LTR), and *GhBURP10* (3 HSEs, 2 TC-rich repeats, 1 MBS and 1 LTR). Similar results were found in the promoters of *MtBURP* [12]. The promoters of 25 *GhBURPs* contained cis-elements involved in defense and stress responsiveness. Heat stress and drought stress elements were more common than other stress-related elements, which suggested that many *GhBURPs*, especially *GhBNM3* with 6 HSEs and *GhRD2* with 5 MBSs, might be involved in heat and drought stress response in cotton. In addition, promoters of 18 *GhBURPs* contained P-box (cis-elements related to GA), indicating that these genes might be involved in the GA regulatory pathway.

### Tissue specific expression patterns of *GhBURPs*

**Fig. 5:**
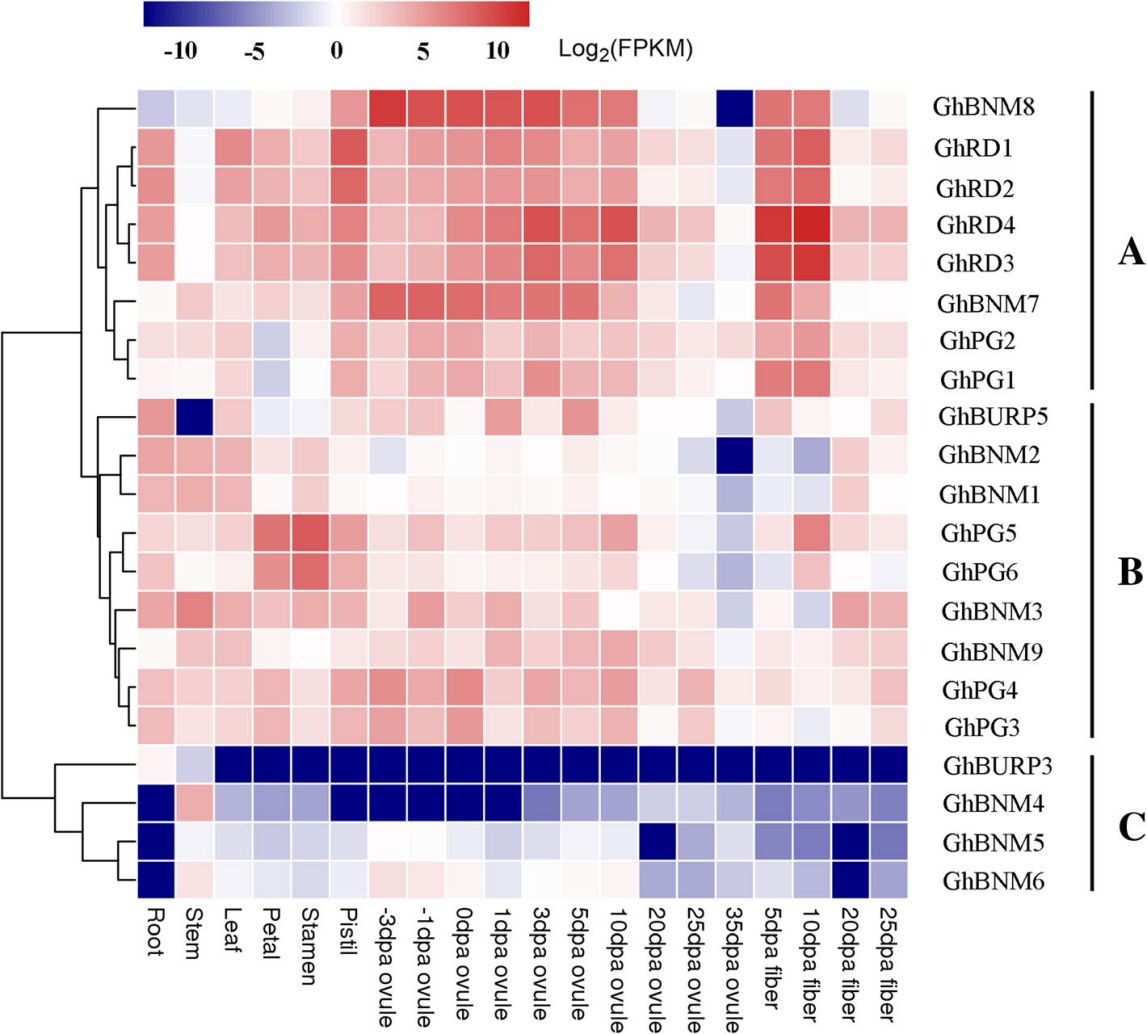
Expression patterns of *GhBURPs* in different tissues. A heatmap indicates the clustering of 21 *GhBURPs* in eight tissues shown on the bottom. The dpa indicates days after anthesis. Gene names are shown on the right. Scale bars at the top show log_2_-transformed FPKM values of each gene

To understand the possible functions of *GhBURPs* during cotton development, we investigated their expression profiles in different cotton organs including root, stem, leaf, petal, stamen, pistil, ovules and fibers using available transcriptomic data [35]. Among the 30 putative *GhBURPs*, 21 *GhBURPs* had a fragments per kilobase of transcript per million fragments (FPKM) ≥ 1 in at least one of the 8 tissues (Fig. 5). The other 9 *GhBURPs*, all from BURP V, showed very low or no expression in the investigated organs, and 7 of them were from gene duplication, which indicated that these genes may be redundancy or pseudogenes, or expressed only in specific tissues at specific developmental stages or in specific environments.

According to the expression features, 21 *GhBURPs* were mainly clustered into three groups (A-C) based on a hierarchical clustering analysis (Fig. 5). Eight *GhBURPs* containing all the members of the RD22-like subfamily, belonging to group A, showed general expression in all the organs and higher expression in reproductive organs (pistil, earlier ovule and fiber) than in other organs. Nine *GhBURPs* belonging to group B showed dominant expression in some organs, such as root, stem, stamen and ovule. The remaining 4 members (*GhBURP3*, *GhBNM5*, *GhBNM4* and *GhBNM6*) belonging to group C showed universal low expression in all the organs except a few organs with slightly higher expression. The different expression patterns might be related to the various functions of *GhBURPs*. *GhRDL1* (*GhRD4*), predominately expressed at the fiber rapid elongation stages (5 and 10 days post anthesis), has been confirmed to promote fiber elongation and increase fruit production by interacting with *GhEXPA1* [30]. *GhBNM8* with preferential expression in ovules, especially at the fiber initiation stage (− 3 DPA). *GhPG5* and *GhPG6* showed dominant expression in stamen, indicating their possible roles during stamen development.

### Stress-induced expression profiles of *GhBURPs*

**Fig. 6:**
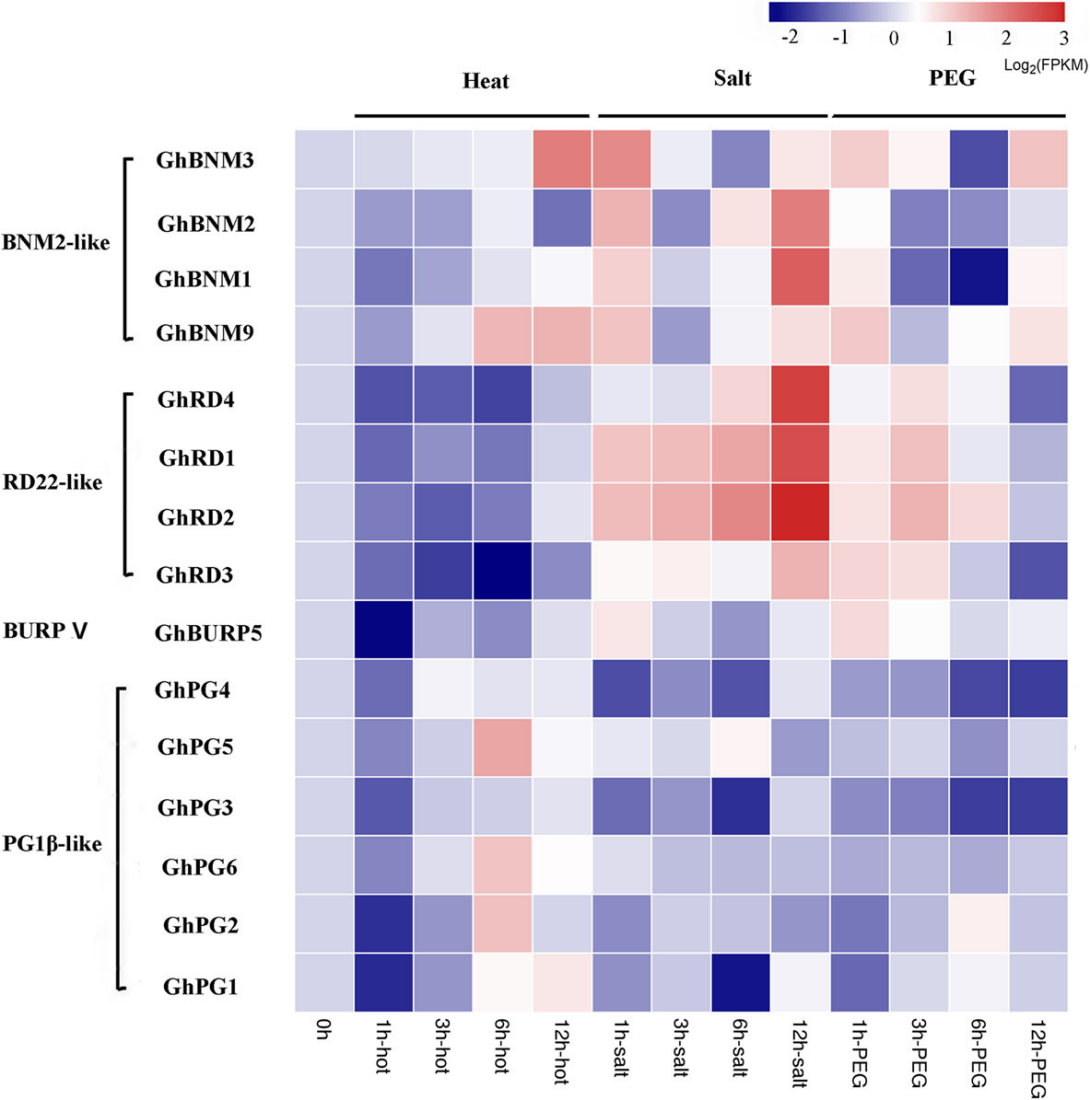
Expression profiles of *GhBURPs* under three stresses. The expression characteristics of 15 GhBURPs under heat, salt and PEG treatments were investigated using available transcriptomic data. 0 h, 1 h, 3 h, 6 h and 12 h indicate hours after different stress treatments. Gene names and the subfamilies are shown on the right. Blocks with colors represent the relative expression levels of *GhBURPs*

According to the analysis of cis-elements in promoter regions and previous studies on *BURPs* in other plants, *GhBURPs* might be involved in stress response. To verify this hypothesis, we analyzed the expression profiles of 15 *GhBURPs* (FPKM ≥1) under heat, salt and drought treatments using available transcriptomic data [35] (Fig. 6). The results revealed that all 15 *GhBURPs* could be induced by all three stresses with different degrees.

Among these genes, *GhBNM3*, a member of the BNM2-like subfamily, was notably induced by heat stress with a continuing increasing trend, which was consistent with the most heat elements in the putative promoter of this gene. Four genes from the RD22-like subfamily, *GhRD1*, *GhRD2*, *GhRD3* and *GhRD4*, with many stress-related elements, especially drought elements, showed significant up-regulated expression under salt and polyethylene glycol (PEG) treatment and down-regulated expression under heat treatment, which accorded with the name “dehydration-responsive protein”. Overall, under heat treatment, most of these genes exhibited early down-regulated and then up-regulated expression. In response to salt and PEG treatments, more than half of *GhBURPs* showed early up-regulated and then down-regulated expression. Similar expression patterns were found among the members of the same subfamilies.

**Fig. 7:**
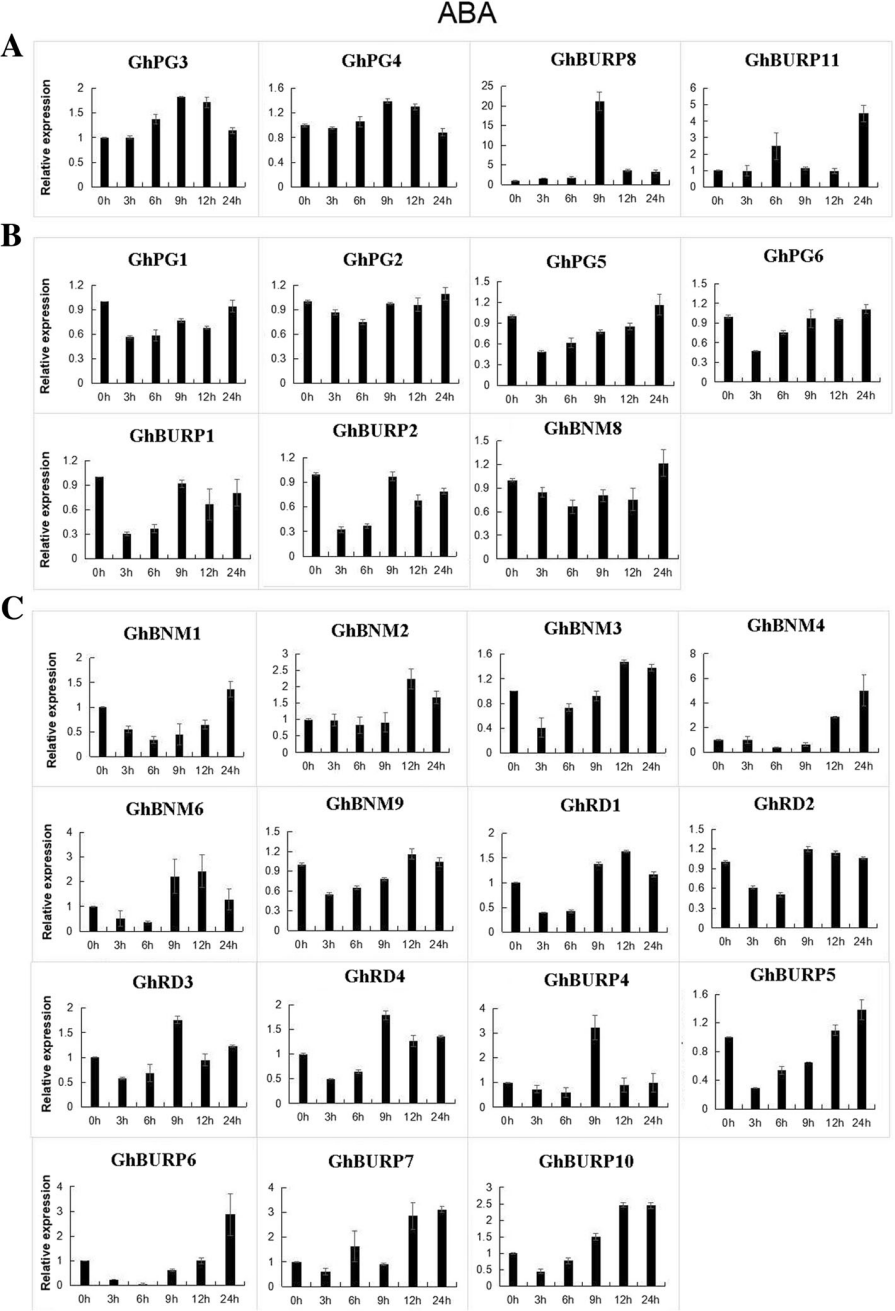
Expression analysis of *GBURPs* under ABA treatments by qRT-PCR. The expression levels of 26 *GhBURPs* were tested by qRT-PCR and estimated by the 2^-△△CT^ method. 0 h, 3 h, 6 h, 9 h, 12 h and 24 h indicate hours after ABA treatment. **a**-**c** represent three expression trends. The error bars show the standard deviation of three biological replicates

**Fig. 8:**
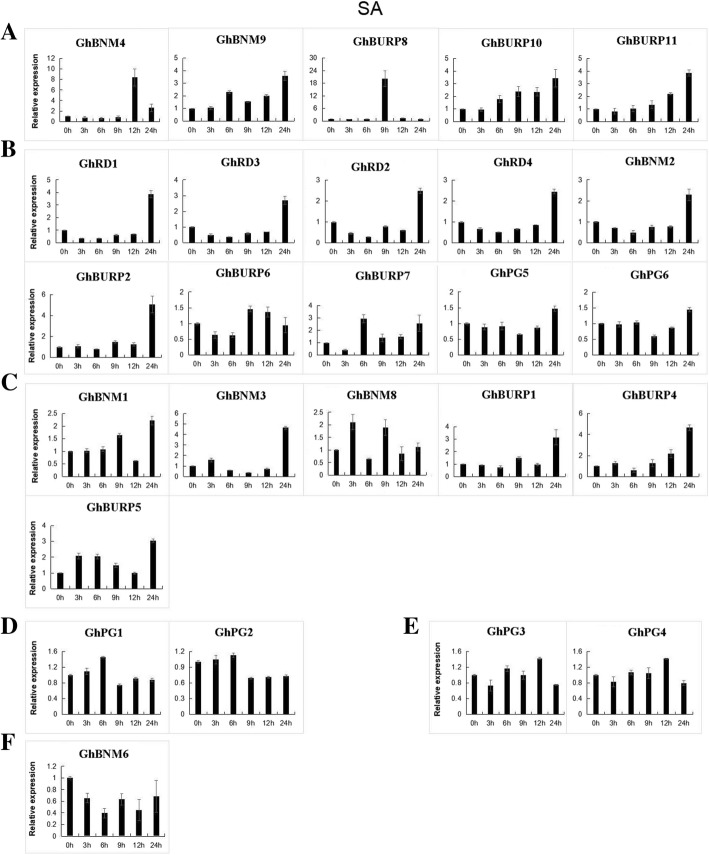
Expression analysis of *GBURPs* under SA treatments by qRT-PCR. The expression levels of 26 *GhBURPs* were tested by qRT-PCR and estimated by the 2^-△△CT^ method. 0 h, 3 h, 6 h, 9 h, 12 h and 24 h indicate hours after SA treatment. **a**-**f** represent six expression trends. The error bars show the standard deviation of three biological replicates

To further explore the potential functions of *GhBURPs* in response to stress-related plant hormones, we also analyzed the expression characteristics of 26 *GhBURPs* (except *GhBURP3*, *GhBNM5*, *GhBNM7* and *GhBURP9* with no detected expression in leaf) under ABA and SA treatment by qRT-PCR (Fig. 7 and Fig. 8). All of the 26 *GhBURPs* were induced by ABA or SA at different points in time after treatments. Under ABA treatment, 26 *GhBURPs* were divided into 3 major patterns (a-c) according to their expression characteristics (Fig. 7). More than half (15/26) of these genes showed an early (mainly at 3 h and 6 h) down-regulation followed by up-regulation (mainly at 12 h and 24 h) (Fig. 7c). Four genes (*GhPG3*, *GhPG4t*, *GhBURP8* and *GhBURP11*) showed up-regulated expression at all time points and reached a maximum at 9 h, except *GhBURP11* at 24 h (Fig. 7a). The expressions of remaining 7 *GhBURPs* were down-regulated with the minimum levels at 3 h and 6 h (Fig. 7b). Under SA treatment, 6 expression patterns were found, and 21 *GhBURPs* were contained in 3 predominant expression patterns: up-regulated expression pattern (5 *GhBURPs*), early down-regulated and then up-regulated expression pattern (10 *GhBURPs*) and early up-regulated, then down-regulated and finally up-regulated expression pattern (6 *GhBURPs*) (Fig. 8). *GhBNM6* showed a down-regulated expression pattern at all time points (Fig. 8f). Two paralogous gene pairs showed two other expression patterns, which were slightly up-regulated and dramatically down-regulated (*GhPG1* / *GhPG2*) (Fig. 8d) and early down-regulated, then up-regulated and finally down-regulated (*GhPG3* / *GhPG4*) (Fig. 8e). In addition, *GhBURP8* and *GhBURP11* showed up-regulated expression at all time points under ABA and SA treatments.

## Discussion

### Characterization of the *BURP* gene family in cotton

*BURP* genes have been extensively studied in many plants for their functions in plant development and responding to stresses [2, 6–8, 22, 26]. The genome-wide analysis of the *BURP* gene family was performed in abundant plants and found different numbers of BURP members. In our study, 18, 17 and 30 BURP genes were identified in *G. raimondii*, *G. arboreum and G. hirsutum*, respectively (Table 1). Recently, two different versions of genome sequences of *G. hirsutum* were obtained using single-molecule real-time sequencing, famous for long-read, which improved the contiguity and completeness for highly repeats chromosomal regions [38, 39]. In the further studies, we will refer to the different genome sequences for more accurate analysis. The analysis of gene duplication events indicated that gene duplication, especially segment duplication, played a significant role in formation of *GhBURP* gene family (Fig. 2 and Table 2) [40, 41].

Whole-genome duplication (WGD) and polyploidization happened throughout the evolutions of many higher plants [42]. For example, in the ancestral seed plant, one WGD occurred ~ 310 MYA and in all eudicots, the paleohexaploidization event occurred ~ 130–190 MYA [43]. In addition, the studies on the evolution of cotton revealed that two WGD events occurred at approximately 115–146 and 13–20 MYA in *Gossypium* [32–34]. Following these two WGD events, A-genome diploids and D-genome diploid began to diverge ~ 5–10 MYA [32, 38]. Then, *G. hirsutum* was formed following the polyploidization of two diploid ancestors at approximately 1–2 MYA [35]. In *G. raimondii*, the divergence time of the segmentally duplicated *BURPs* was inferred to be ~ 264 MYA, indicating that the segmentally duplicated *BURPs* began to divergence before the ancestral WGD. The divergence time of tandem duplicated *BURPs* in *G. raimondii* and *G. arboreum* were inferred to be 8 and 19 MYA, which were after two WGD events. In *G. hirsutum*, the inferred divergence times of segmentally duplicated *GhBURPs* were ~ 5.65 to 26.30 MYA, indicating that the divergence of the segmentally duplicated *GhBURPs* were accompanied with the divergence of A and D progenitor genomes (Table 2). As an indicator of selection pressure on a gene or gene region, the analysis of ratios of Ka/Ks indicated that these gene duplication pairs performed similar functions for limitation of purifying selection in function divergence [44]. Based on the analysis of similarity and synteny, some homologs of *GrBURPs* and *GaBURPs* were missing in *G. hirsutum*, which might be due to gene loss caused by deletion or rearrangement of segment in chromosomes, point mutations and insertion or deletion of genes during the evolutional history of *G. hirsutum* [43].

The results of phylogenetic analysis showed that the BURP members from nine species could be classified into eight subfamilies (Fig. 1 and Additional file 1: Table S1). The RD22-like, PG1β-like and BURP VIII subfamilies were composed of BURP members from monocots and dicots, indicating that these genes might have originated before the divergence of monocots and dicots [10, 12]. However, the members of other subfamilies were only from investigated monocots (BURP VI and BURP VII) or dicots (USP-like, BNM2-like and BURP V subfamilies), indicating that these genes might evolve separately and perform different functions between monocots and dicots [9, 13, 45]. Some results were different from previous studies due to the different methods and species used in the phylogenetic analysis. In our study, we searched BURP proteins in the more species including two monocots, seven dicots and four host BURP proteins than previous studies and 1000 bootstrap replications were used for more reliable classification results in phylogenetic analysis.

According to the structure analysis, all 65 BURP proteins contained conserved BURP-domain, especially the four notable CH motifs (Additional file 4: Figure S1 and Additional file 5: Figure S2). The component motifs and arrangement of motifs of members were conserved in the same subfamilies and were diverse among different subfamilies, which was in accordance with the results of the exon-intron structure of *BURPs* (Fig. 3). Similar results were found in previous studies and might be related to *BURPs’* conserved and different functions in plant development, metabolism and stress resistance [9, 10, 13, 21].

### Putative functions of *GhBURPs*

Gene expression patterns were usually known as a significant indication to explore their functions [46]. To explore the potential functions of *GhBURPs*, their expression patterns in eight tissues were investigated (Fig. 5). The results revealed that the paralogous gene pairs showed similar expression characteristics, indicating that these genes may execute analogical functions, which was confirmed by the expression patterns of *BURPs* in *Glycine max* [10]. Notably, *GhBNM7* and *GhBNM8*, homologous to *AtUSPL1*, which regulated seed development [21], were dominantly expressed in early-stage ovules (cotton seed), hinting their potential roles in ovule and fiber development. *GhPG5* and *GhPG6,* homologous to *AtPGLs*, were preferentially expressed in stamen [22, 47], indicating their functions in pollen development possibly by degrading pectin of the cell wall. In addition, *GhRD1*, *GhRD2 GhRD3*, and *GhRD4*, homologous to *AtRD22*, were pervasively expressed in the investigated organs, especially in fiber, and had higher expression levels in root than other members of the *GhBURP* gene family. Meanwhile, previous studies have attested that *GhRD4* regulated fruit production and fiber length through controlling cell wall expansion and *RD22-like* genes could respond to dehydration and other stresses [2, 30, 31, 46, 48]. These results indicated that these genes might play potential roles in fiber development and the response to various stresses, which needs to be further verified.

Since the “R” gene (*AtRD22*) of “BURP” was identified, the stress response of *BURP* genes gradually became an indisputable fact [2]. The analysis of cis-elements revealed that all putative promoters of the 30 *GhBURP* contained at least one of the investigated stress-responsive elements, suggesting that these genes might respond to various stresses (Fig. 4 and Additional file 7: Table S5). Notably, most (25/30) of *GhBURPs* contained defense and stress response-related cis-elements, indicating that the functions involved in stress responses were maintained during evolution of the *GhBURP* gene family. Consistently, expression patterns of 15 *GhBURPs* under three stress treatments (heat, salt and PEG) showed that all of these genes responded to stress treatments at different levels (Fig. 6). Among them, the expression of *GhBNM3* with 10 stress-related cis-elements and four *RD22-like* genes (*GhRD1*, *GhRD2 GhRD3* and *GhRD4*) with 4 to 8 stress-related cis-elements were remarkably up-regulated under heat and salt stress treatments, respectively, indicating that these genes might respond to heat and salt stresses via stress-related cis-elements.

Previous studies have indicated that the ABA and SA signal pathway were important to stress responses and plant defense [9, 49]. Some *BURP* genes including *BnBDC1* [25], *AtRD22* [23], two *OsBURPs* [9] and fifteen *BURPs* in *Glycine max* [10] were induced by ABA. In our study, qRT-PCR analyses revealed that 26 *GhBURPs* were induced by ABA and SA at different points, indicating that this gene family probably participate in ABA or SA signaling pathways (Figs. 7 and 8). The four members from the RD22-like subfamily showed similar expression pattern (initially down-regulated and later up-regulated) in response to ABA and SA. In addition, *GhBURP8* and *GhBURP11*, with neither ABA- nor SA- responsive elements, had a strongly up-regulated expression pattern, indicating that some unidentified stress-related cis-elements might be crucial to response and defense to stresses in cotton.

In general, most *GhBURPs* (except genes with very low or no expression in investigated tissues) were induced by heat, salt, PEG, ABA and SA, even though the induction of some *GhBURPs* was slight. Similar to previous reports, the members of the RD22-like subfamily (*GhRD1*, *GhRD2 GhRD3* and *GhRD4*) were clearly induced by stress treatments, especially by heat and salt treatments, and might be related to the higher expression in root than other members [2]. Meanwhile, the significant roles of *GhRD4* in regulating fruit production and fiber length have been proved in previous studies [30, 31], implying that this gene executed important functions in plant growth and response to abiotic stresses. The other three genes showed expression profiles similar to *GhRD4* in investigated organs and under stress treatments indicating their similar functions. The expression levels of *GhBURPs* from other subfamilies also changed under stresses, which was similar to the result in *Medicago* [12]. Taken together, *GhBURPs* were extensively induced by stresses, revealing that these genes perform important functions in response to different stresses in cotton. These results implied *GhBURP* gene family’s potential importance in improving cotton stress tolerance.

## Conclusion

In this study, a systematical analysis was carried out to investigate the BURP domain-containing gene family in three cotton species (*G. raimondii*, *G. arboreum* and *G. hirsutum*). Based on analyses of phylogeny, gene structure and conserved motifs, the *BURP* genes could be divided into 8 subfamilies. Gene duplication analysis indicated that *GhBURP* gene family expansion might be due to segmental duplication. Expression analysis revealed that *GhBURPs* exhibited different expression characteristics at different organs. The analyses of qRT-PCR and transcriptomic data indicated that *GhBURPs* were induced by various stresses. The results of our study provide a comprehensive view of the *GhBURP* gene family and might be valuable for improving of cotton stress tolerance.

## Methods

### Identification of *BURPs* in cotton

The hidden markov model (HMM) profile corresponding to the BURP domain (PF03181) domain was downloaded from Pfam database (http://pfam.xfam.org) [50]. The genomic databases of *G. raimondii*, *G. arboreum* and *G. hirsutum* were obtained from the CottonGen website (https://www.cottongen.org/icgi/home), The genomes of other species were downloaded from phytozome database (https://phytozome.jgi.doe.gov/). HMMER 3.0 was used to search the *BURP* genes from the three cotton species genomes [51]. The default parameter of e-value threshold was set at 1e− 10. Then, all of the candidate BURP proteins acquired from the HMM search, further confirmed the existence of the BURP domain using SMART database (http://smart.embl-heidelberg.de/) with the normal mode [52]. The *BURP* genes in other species were identified with the same method.

The Mw, pI and GRAVY of the identified BURP proteins were predicted using ExPASy website (http://web.expasy.org/protparam/) and the subcellular localization was predicted through CELLO v2.5 server (http://cello.life.nctu.edu.tw/) [53, 54].

### Multiple sequence alignments and phylogenetic analysis

All identified BURP protein sequences from *G. raimondii*, *G. arboreum*, *G. hirsutum*, *Arabidopsis*, *Glycine max*, *Theobroma cacao*, *Oryza sativa*, *Populus trichocarpa*, *Oryza sativa*, *Zea mays* and 4 host BURP members (AtRD22, VfUSPs, BNM2 and Le-PG1β) were aligned using ClustalX 2.0 [55]. The phylogenetic tree was constructed using the neighbor-joining (NJ) method of MEGA 6.0 with the p-distance model and 1000 bootstrap replications [56].

### Chromosomal location, gene duplication and syntenic analysis of *BURP* genes

The distribution of *BURP* genes was determined by MapInspect (http://www.plantbreeding.wur.nl/uk/ software-mapinspect.html) based on physical location from genome databases of three cotton species. First, the predicted BURP proteins were aligned using ClustalW2 at EMBL-EBI (http://www.ebi.ac.uk/Tools/msa/clustalw2/). Then, gene duplication events were determined according to the following criteria: (1) the coverage of alignment sequence was ≥80% of the longer gene; (2) the similarity of the aligned regions was ≥80%; and (3) tightly linked genes on the same chromosome were considered as tandem duplication [37, 57]. The syntenic analysis was performed by using MCScanX software with the default parameters [58]. Circos was adopted to plot the diagram of segmental duplication events on chromosomes [59].

The selection pressure of each BURP gene duplication event was calculated by Ka/Ks rates using DnaSp V5.0 software [60]. Generally, Ka/Ks < 1 signified purifying selection; Ka/Ks = 1 signified neutral selection; and Ka/Ks > 1 signified positive selection. The divergence times of these gene duplication pairs were speculated according to previous reports [38, 61].

### Sequence analysis

Multiple sequence alignment was performed using identified BURP proteins with ClustalX 2.0. The locations of conserved BURP domains and potential signal peptides of these proteins were determined using SMART database and SignalP 4.0 server (http://www.cbs.dtu.dk/services/SignalP/), respectively [45, 62].

The organization of exon-intron structures of *BURP* genes was ascertained by comparing coding sequences with corresponding full-length genes on the Gene Structure Display Server (GSDS: http://gsds.cbi.pku.edu.cn/) [63]. The conserved motifs from BURP proteins were identified via MEME program. The optimized parameters were set as follows: size distribution, zero or one occurrence per sequence; motif count, 15; and motif width, between 6 and 50 residues [64].

### Cis-elements in the promoter region

The regulatory elements in the promoter regions were predicated using PlantCARE database (http://bioinformatics.psb.ugent.be/webtools/plantcare/html/) [31, 65].

### Plant materials and treatments

In this study, *G. hirsutum* L. acc. Texas Marker-1 (TM-1) was grown in a climate-controlled chamber (light/dark cycle: 16 h at 28 °C/8 h at 22 °C). Four-week-old seedlings with exhibiting third true leaves were treated with 100 μM ABA, 100 μM SA and water as a control group. The leaves were collected at 0 h, 3 h, 6 h, 9 h, 12 h and 24 h after treatments. After harvest, all the treated materials were immediately frozen in liquid nitrogen and stored at − 80 °C.

### RNA extraction and gene expression analysis

Total RNA was isolated from the collected samples using the RNA-prep Pure Plant Kit (TIANGEN, Beijing, China). The DNA-free RNA was reverse transcribed using the PrimeScript RT Reagent kit (Takara, Dalian, China) according to the manufacturer’s instructions. qRT-PCR was carried out with an ABI 7500 Real-Time PCR System (Applied Biosystems) using SYBR Premix Ex Taq (Takara). The protocol was performed as follows: step 1: 95 °C for 30 s; step 2: 40 cycles of 95 °C for 5 s, 60 °C for 34 s; and step 3: melting curve analysis. Three biological replicates and the 2^-△△CT^ method were used to calculate the relative expression levels of *GhBURPs* [66]. The cotton histone3 gene (GeneBank accession no. AF024716) was used as an internal reference gene to normalize target gene expression levels [57]. The gene-specific primers used for qRT-PCR were designed by Primer 5.0 (Additional file 8: Table S6).

The expression levels of *GhBURPs* in different tissues and under treatments (heat, salt and drought) were investigated using the reported transcriptomic data [35].

## Additional files

Additional file 1: **Table S1.**. Numbers of BURPs in the eight subfamilies from different species. (XLSX 10 kb)

Additional file 2: **Table S2.**. Orthologous gene pairs of *G. hirsutum*, *G. arboreum* and *G. raimondii*. (XLSX 10 kb)

Additional file 3: **Table S3.**. Location of the BURP domain and signal peptide in BURP proteins. (XLSX 12 kb)

Additional file 4: **Figure S1.** The conserved BURP domain and signal peptide of BURP proteins in three cotton species. A: Phylogenetic analysis of 65 BURP proteins and subfamilies of these proteins are shown using MEGA 6.0 with the neighbor-joining (NJ) method. B: Conserved domains of 65 BURP proteins. The red filled boxes represent the BURP domain, and the green filled boxes represent the signal peptide. (TIF 9369 kb)

Additional file 5: **Figure S2.** Conserved motifs and amino acid sites in the BURP domain. Multiple alignment analysis of 65 BURP proteins using ClustalX 2.0. The BURP domain contained seven motifs (motif 1, 2, 3, 4, 5, 6 and 7). The red, blue and green asterisks represent 4 conserved CH motifs, two phenylalanine (F) and two cysteine (C) sites and one highly conserved aspartic acid (D) found in these proteins, respectively. (TIF 9215 kb)

Additional file 6: **Table S4.** Numbers of introns and exons of *BURPs*. (XLSX 12 kb)

Additional file 7: **Table S5.** Number of cis-elements in putative promoters of *GhBURPs*. (XLSX 12 kb)

Additional file 8: **Table S6.** Primer pairs used in this investigation. (XLSX 12 kb)

## Data Availability

The data included in this article and the additional files are available. The transcriptome datasets of *G. hirsutum* TM-1 are under the accession number PRJNA248163 in NCBI.

## Abbreviations

AA: amino acid
ABA: abscisic acid
ABRE: abscisic acid responsive element
BURP: BNM2, USP, RD22 and PG1β
DPA: days post anthesis
ERE: ethylene-responsive element
FPKM: fragments per kilobase of transcript per million fragments
GA: gibberellin
*Ga*: *Gossypium arboreum*
*Gh*: *Gossypium hirsutum*
*Gr*: *Gossypium raimondii*
HMM: hidden markov model
HSE: heat stress-responsive element
Ka: nonsynonymous substitution rate
Ks: synonymous substitution rate
MBS: MYB binding site involved in drought- inducibility
MeJA: methyl jasmonate
MYA: million years ago
qRT-PCR: quantitative real-time polymerase chain reaction
SA: salicylic acid
WGD: whole-genome duplication

## References

[1] He L, Yang X, Wang L, Zhu L, Zhou T, Deng J, Zhang X (2013). Molecular cloning and functional characterization of a novel cotton CBL-interacting protein kinase gene (GhCIPK6) reveals its involvement in multiple abiotic stress tolerance in transgenic plants. Biochem Biophys Res Commun.

[2] Yamaguchi-Shinozaki K, Shinozaki K (1993). The plant hormone abscisic acid mediates the drought-induced expression but not the seed-specific expression of rd22, a gene responsive to dehydration stress in Arabidopsis thaliana. Mol Gen Genet.

[3] Jeon J-S, Chung Y-Y, Lee S, Yi G-H, Oh B-G, An G (1999). Isolation and characterization of an anther-specific gene, RA8, from rice (Oryza sativa L.). Plant Mol Biol.

[4] Phillips K, Ludidi N (2017). Drought and exogenous abscisic acid alter hydrogen peroxide accumulation and differentially regulate the expression of two maize RD22-like genes. Sci Rep.

[5] Boutilier KA, Gines MJ, DeMoor JM, Huang B, Baszczynski CL, Lyer VN, Miki BL (1994). Expression of the BnmNAP subfamily of napin genes coincides with the induction of Brassica microspore embryogenesis. Plant Mol Biol.

[6] Bassüner R, Bäumlein H, Huth A, Jung R, Wobus U, Rapoport TA, Saalbach G, Müntz K (1988). Abundant embryonic mRNA in field bean(Vicia faba L.) codes for a new class of seed proteins: cDNA cloning and characterization of the primary translation product. Plant Mol Biol.

[7] Zheng L, Heupel RC, DellaPenna D (1992). The beta subunit of tomato fruit polygalacturonase isoenzyme 1: isolation, characterization, and identification of unique structural features. Plant Cell.

[8] Hattori J, Boutilier KA, van Lookeren Campagne MM, Miki BL (1998). A conserved BURP domain defines a novel group of plant proteins with unusual primary structure. Mol Gen Genet.

[9] Ding X, Hou X, Xie K, Xiong L (2009). Genome-wide identification of BURP domain-containing genes in rice reveals a gene family with diverse structures and responses to abiotic stresses. Planta.

[10] Xu H, Li Y, Yan Y, Wang K, Gao Y, Hu Y (2010). Genome-scale identification of soybean BURP domain-containing genes and their expression under stress treatments. BMC Plant Biol.

[11] Shao Y, Wei G, Wang L, Dong Q, Zhao Y, Chen B, Xiang Y (2011). Genome-wide analysis of BURP domain-containing genes in Populus trichocarpa. J Integr Plant Biol.

[12] Li Y, Chen X, Chen Z, Cai R, Zhang H, Xiang Y (2016). Identification and expression analysis of BURP domain-containing genes in Medicago truncatula. Front Plant Sci.

[13] Gan D, Jiang H, Zhang J, Zhao Y, Zhu S, Cheng B (2011). Genome-wide analysis of BURP domain-containing genes in maize and sorghum. Mol Biol Rep.

[14] Granger C, Coryell V, Khanna A, Keim P, Vodkin L, Shoemaker RC (2002). Identification, structure, and differential expression of members of a BURP domain containing protein family in soybean. Genome.

[15] Treacy BK, Hattori J, Prud’homme I, Barbour E, Boutilier K, Baszczynski CL, Huang B, Johnson DA, Miki BL (1997). Bnm1, a Brassica pollen-specific gene. Plant Mol Biol.

[16] Teerawanichpan P, Xia Q, Caldwell SJ, Datla R, Selvaraj G (2009). Protein storage vacuoles of Brassica napus zygotic embryos accumulate a BURP domain protein and perturbation of its production distorts the PSV. Plant Mol Biol.

[17] Chesnokov Y, Meister A, Manteuffel R (2002). A chimeric green fluorescent protein gene as an embryonic marker in transgenic cell culture of Nicotiana plumbaginifolia Viv. Plant Sci.

[18] Chen L, Miyazaki C, Kojima A, Saito A, Adachi A (1999). Isolation and characterization of a gene expressed during early embryo sac development in apomictic Guinea grass (Panicum maximum). Plant Physiol.

[19] Wang A, Xia Q, Xie W, Datla R, Selvaraj G (2003). The classical Ubisch bodies carry a sporophytically produced structural protein (RAFTIN) that is essential for pollen development. Proc Natl Acad Sci U S A.

[20] Batchelor AK, Boutilier K, Miller SS, Hattori J, Bowman LA, Hu M, Lantin S, Johnson DA, Miki BL (2002). SCB1, a BURP-domain protein gene, from developing soybean seed coats. Planta.

[21] Van Son L, Tiedemann J, Rutten T, Hillmer S, Hinz G, Zank T, Manteuffel R, Baumlein H (2009). The BURP domain protein AtUSPL1 of Arabidopsis thaliana is destined to the protein storage vacuoles and overexpression of the cognate gene distorts seed development. Plant Mol Biol.

[22] Watson CF, Zheng L, DellaPenna D (1994). Reduction of tomato polygalacturonase beta subunit expression affects pectin solubilization and degradation during fruit ripening. Plant Cell.

[23] Abe H, Yamaguchi-Shinozaki K, Urao T, Iwasaki T, Hosokawa D, Shinozaki K (1997). Role of Arabidopsis MYC and MYB homologs in drought- and abscisic acid-regulated gene expression. Plant Cell.

[24] Banzai T, Sumiya K, Hanagata N, Dubinsky Z, Karube I (2001). Molecular cloning and characterization of genes encoding BURP domain-containing protein in the mangrove, Bruguiera gymnorrhiza. Trees.

[25] Shunwu Y, Zhang L, Zuo K, Li Z, Tang K (2004). Isolation and characterization of a BURP domain-containing gene BnBDC1 from Brassica napus involved in abiotic and biotic stress. Physiol Plant.

[26] Almazroue, HA: Identification, cloning, and expression of tobacco responsive to dehydration like protein (RD22), BIP-355 and its role in SABP2 mediated SA pathway in plant defense. Electronic Theses and Dissertations 2014, 2456.

[27] Ragland M, Soliman KM. Sali5-4a and Sali3-2, two genes induced by aluminum in soybean roots. Plant Physiol. 1997;114:395.

[28] Tang Y-L, Li X-J, Zhong Y-T, Zhang Y-Z (2007). Functional analysis of soybean SALI3-2 in yeast. J Shenzhen Univ Sci Eng.

[29] Datta N, LaFayette PR, Kroner PA, Nagao RT, Key JL (1993). Isolation and characterization of three families of auxin down-regulated cDNA clones. Plant Mol Biol.

[30] Xu B, Gou JY, Li FG, Shangguan XX, Zhao B, Yang CQ, Wang LJ, Yuan S, Liu CJ, Chen XY (2013). A cotton BURP domain protein interacts with alpha-expansin and their co-expression promotes plant growth and fruit production. Mol Plant.

[31] Shan CM, Shangguan XX, Zhao B, Zhang XF, Chao LM, Yang CQ, Wang LJ, Zhu HY, Zeng YD, Guo WZ (2014). Control of cotton fibre elongation by a homeodomain transcription factor GhHOX3. Nat Commun.

[32] Paterson AH, Wendel JF, Gundlach H, Guo H, Jenkins J, Jin D, Llewellyn D, Showmaker KC, Shu S, Udall J (2012). Repeated polyploidization of Gossypium genomes and the evolution of spinnable cotton fibres. Nature.

[33] Wang K, Wang Z, Li F, Ye W, Wang J, Song G, Yue Z, Cong L, Shang H, Zhu S (2012). The draft genome of a diploid cotton Gossypium raimondii. Nat Genet.

[34] Li F, Fan G, Wang K, Sun F, Yuan Y, Song G, Li Q, Ma Z, Lu C, Zou C (2014). Genome sequence of the cultivated cotton Gossypium arboreum. Nat Genet.

[35] Zhang T, Hu Y, Jiang W, Fang L, Guan X, Chen J, Zhang J, Saski CA, Scheffler BE, Stelly DM (2015). Sequencing of allotetraploid cotton (Gossypium hirsutum L. acc. TM-1) provides a resource for fiber improvement. Nat Biotechnol.

[36] Li F, Fan G, Lu C, Xiao G, Zou C, Kohel RJ, Ma Z, Shang H, Ma X, Wu J (2015). Genome sequence of cultivated upland cotton (Gossypium hirsutum TM-1) provides insights into genome evolution. Nat Biotechnol.

[37] Innan H, Kondrashov F (2010). The evolution of gene duplications: classifying and distinguishing between models. Nat Rev Genet.

[38] Hu Y, Chen J, Fang L, Zhang Z, Ma W, Niu Y, Ju L, Deng J, Zhao T, Lian J (2019). Gossypium barbadense and Gossypium hirsutum genomes provide insights into the origin and evolution of allotetraploid cotton. Nat Genet.

[39] Wang M, Tu L, Yuan D, Zhu SC, Li J, Liu F, Pei L, Wang P, Zhao G (2019). Reference genome sequences of two cultivated allotetraploid cottons, Gossypium hirsutum and Gossypium barbadense. Nat Genet.

[40] Flagel LE, Wendel JF (2009). Gene duplication and evolutionary novelty in plants. The New phytologist.

[41] Pickett FB, Meeks-wanger DR (1995). Seeing double: appreciating genetic redundancy. Plant Cell.

[42] Jackson S, Chen ZJ (2010). Genomic and expression plasticity of polyploidy. Curr Opin Plant Biol.

[43] Wang W, Zhang X, Deng F, Yuan R, Shen F (2017). Genome-wide characterization and expression analyses of superoxide dismutase (SOD) genes in Gossypium hirsutum. BMC Genomics.

[44] Lynch M, Conery J (2000). The evolutionary fate and consequences of duplicate genes. Science.

[45] Sun H, Hao P, Ma Q, Zhang M, Qin Y, Wei H, Su J, Wang H, Gu L, Wang N (2018). Genome-wide identification and expression analyses of the pectate lyase (PEL) gene family in cotton (Gossypium hirsutum L.). BMC Genomics.

[46] Matus JT, Aquea F, Espinoza C, Vega A, Cavallini E, Dal Santo S, Canon P (2014). Rodriguez-Hoces de la Guardia a, serrano J, Tornielli GB *et al*: inspection of the grapevine BURP superfamily highlights an expansion of RD22 genes with distinctive expression features in berry development and ABA-mediated stress responses. PLoS One.

[47] Park J, Cui Y, Kang BH (2015). AtPGL3 is an Arabidopsis BURP domain protein that is localized to the cell wall and promotes cell enlargement. Front Plant Sci.

[48] Wang H, Zhou L, Fu Y, Cheung MY, Wong FL, Phang TH, Sun Z, Lam HM (2012). Expression of an apoplast-localized BURP-domain protein from soybean (GmRD22) enhances tolerance towards abiotic stress. Plant Cell Environ.

[49] Shah J (2003). The salicylic acid loop in plant defense. Curr Opin Plant Biol.

[50] Finn RD, Coggill P, Eberhardt RY, Eddy SR, Mistry J, Mitchell AL, Potter SC, Punta M, Qureshi M, Sangrador-Vegas A (2016). The Pfam protein families database: towards a more sustainable future. Nucleic Acids Res.

[51] Yu J, Jung S, Cheng CH, Ficklin SP, Lee T, Zheng P, Jones D, Percy RG, Main D (2014). CottonGen: a genomics, genetics and breeding database for cotton research. Nucleic Acids Res.

[52] Letunic I, Doerks T, Bork P (2015). SMART: recent updates, new developments and status in 2015. Nucleic Acids Res.

[53] Artimo P, Jonnalagedda M, Arnold K, Baratin D, Csardi G, de Castro E, Duvaud S, Flegel V, Fortier A, Gasteiger E (2012). ExPASy: SIB bioinformatics resource portal. Nucleic Acids Res.

[54] Yu CS, Lin CJ, Hwang JK (2004). Predicting subcellular localization of proteins for gram-negative bacteria by support vector machines based on n-peptide compositions. Protein Sci.

[55] Li X, Liu G, Geng Y, Wu M, Pei W, Zhai H, Zang X, Li X, Zhang J, Yu S (2017). A genome-wide analysis of the small auxin-up RNA (SAUR) gene family in cotton. BMC Genomics.

[56] Tamura K, Stecher G, Peterson D, Filipski A, Kumar S (2013). MEGA6: molecular evolutionary genetics analysis version 6.0. Mol Biol Evol.

[57] Liu Z, Ge X, Yang Z, Zhang C, Zhao G, Chen E, Liu J, Zhang X, Li F (2017). Genome-wide identification and characterization of SnRK2 gene family in cotton (Gossypium hirsutum L.). BMC Genet.

[58] Wang Y, Tang H, Debarry JD, Tan X, Li J, Wang X, Lee TH, Jin H, Marler B, Guo H (2012). MCScanX: a toolkit for detection and evolutionary analysis of gene synteny and collinearity. Nucleic Acids Res.

[59] Krzywinski M, Schein J, Birol I, Connors J, Gascoyne R, Horsman D, Jones SJ, Marra MA (2009). Circos: an information aesthetic for comparative genomics. Genome Res.

[60] Librado P, Rozas J (2009). DnaSP v5: a software for comprehensive analysis of DNA polymorphism data. Bioinformatics.

[61] Li F, Fan K, Ma F, Yue E, Bibi N, Wang M, Shen H, Hasan MM, Wang X (2016). Genomic identification and comparative expansion analysis of the non-specific lipid transfer protein gene family in Gossypium. Sci Rep.

[62] Petersen TN, Brunak S, von Heijne G, Nielsen H (2011). SignalP 4.0: discriminating signal peptides from transmembrane regions. Nat Methods.

[63] Hu B, Jin J, Guo A-Y, Zhang H, Luo J, Gao G (2015). GSDS 2.0: an upgraded gene feature visualization server. Bioinformatics.

[64] Bailey TL, Williams N, Misleh C, Li WW (2006). MEME: discovering and analyzing DNA and protein sequence motifs. Nucleic Acids Res.

[65] Lescot M, Déhais P, Thijs G, Marchal K, Moreau Y, Peer YV, Rouzé P, Rombauts S (2002). PlantCARE, a database of plant cis-acting regulatory elements and a portal to tools for in silico analysis of promoter sequences. Nucleic Acids Res.

[66] Livak KJ, Schmittgen TD (2001). Analysis of relative gene expression data using real-time quantitative PCR and the 2(−Delta Delta C(T)) method. Methods.

