# Supplementary figures and images for: Genome-wide identification and expression analysis of the BURP domain-containing genes in Gossypium hirsutum
Source: BMC Genomics. 2019 Jul 8;20:558. doi: 10.1186/s12864-019-5948-y (PMC6615115; doi:10.1186/s12864-019-5948-y)

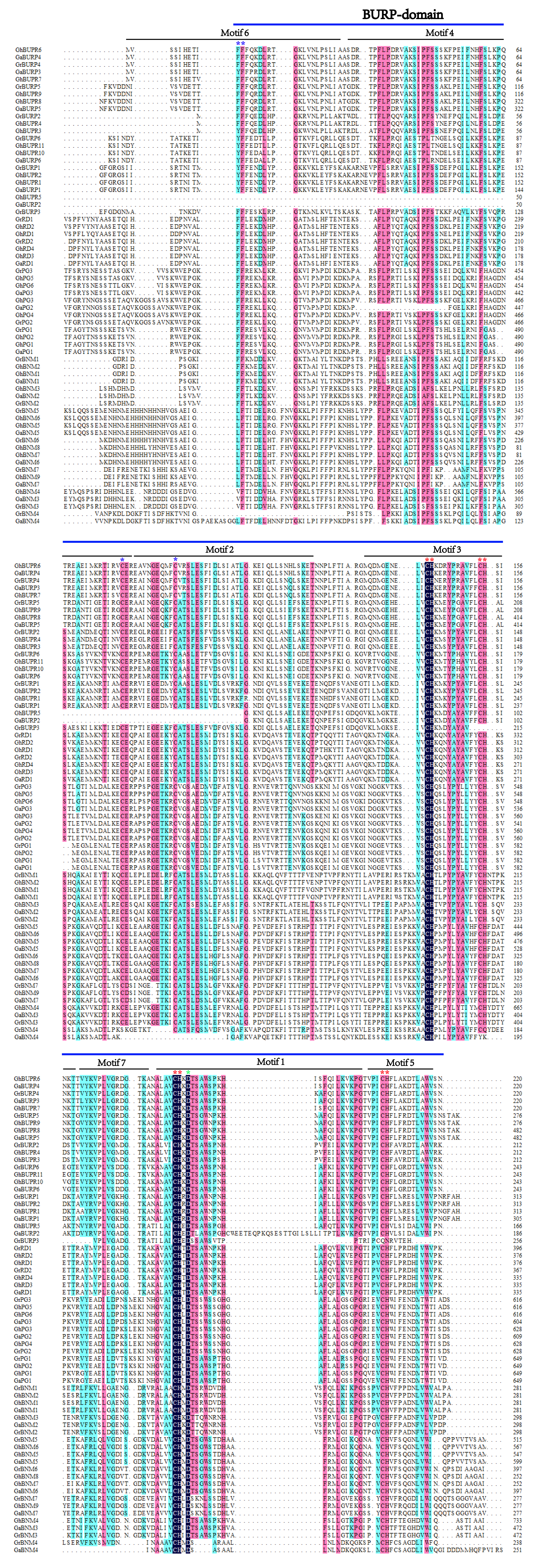

Supplement: Supplementary file 5 — Figure S2. Conserved motifs and amino acid sites in the BURP domain. Multiple alignment analysis of 65 BURP proteins using ClustalX 2.0. The BURP domain contained seven motifs (motif 1, 2, 3, 4, 5, 6 and 7). The red, blue and green asterisks represent 4 conserved CH motifs, two phenylalanine (F) and two cysteine (C) sites and one highly conserved aspartic acid (D) found in these proteins, respectively. (TIF 9215 kb) [file 12864_2019_5948_MOESM5_ESM.tif]
